# Identification of oncogenic driver mutations by genome-wide CRISPR-Cas9 dropout screening

**DOI:** 10.1186/s12864-016-3042-2

**Published:** 2016-09-09

**Authors:** Michael K. Kiessling, Sven Schuierer, Silke Stertz, Martin Beibel, Sebastian Bergling, Judith Knehr, Walter Carbone, Cheryl de Vallière, Joelle Tchinda, Tewis Bouwmeester, Klaus Seuwen, Gerhard Rogler, Guglielmo Roma

**Affiliations:** 1 Department of Gastroenterology and Hepatology, University Hospital Zürich, Zürich, Switzerland; 2 Novartis Institutes for Biomedical Research, Novartis Pharma AG, Basel, Switzerland; 3 Institute of Medical Virology, University of Zürich, Zürich, Switzerland; 4 Department of Oncology, Children University Hospital Zürich, Zürich, Switzerland

**Keywords:** Whole genome CRISPR screen, Dropout, Negative selection, Driver mutations, EGFR, NRAS, Kinase

## Abstract

**Background:**

Genome-wide CRISPR-Cas9 dropout screens can identify genes whose knockout affects cell viability. Recent CRISPR screens detected thousands of essential genes required for cellular survival and key cellular processes; however discovering novel lineage-specific genetic dependencies from the many hits still remains a challenge.

**Results:**

To assess whether CRISPR-Cas9 dropout screens can help identify cancer dependencies, we screened two human cancer cell lines carrying known and distinct oncogenic mutations using a genome-wide sgRNA library. We found that the gRNA targeting the driver mutation EGFR was one of the highest-ranking candidates in the EGFR-mutant HCC-827 lung adenocarcinoma cell line. Likewise, sgRNAs for NRAS and MAP2K1 (MEK1), a downstream kinase of mutant NRAS, were identified among the top hits in the NRAS-mutant neuroblastoma cell line CHP-212. Depletion of these genes targeted by the sgRNAs strongly correlated with the sensitivity to specific kinase inhibitors of the EGFR or RAS pathway in cell viability assays. In addition, we describe other dependencies such as TBK1 in HCC-827 cells and TRIB2 in CHP-212 cells which merit further investigation.

**Conclusions:**

We show that genome-wide CRISPR dropout screens are suitable for the identification of oncogenic drivers and other essential genes.

**Electronic supplementary material:**

The online version of this article (doi:10.1186/s12864-016-3042-2) contains supplementary material, which is available to authorized users.

## Background

The RNA-guided CRISPR (clustered regularly interspaced short palindrome repeats)-associated nuclease Cas9 has become a powerful and versatile tool to study the functional relevance of genes in biological processes and disease settings [1–7]. Cas9 and a single guide RNA (sgRNA) cause DNA double-strand breaks (DSB) at specific genomic sites, targeted by the sgRNA sequence [8, 9]. DSBs are repaired by the nonhomologous end-joining (NHEJ) pathway generating short insertions and deletions (indels), which ultimately may result in a loss-of-function allele [8, 9].

Whole-genome screens by CRISPR-Cas9 can be deployed in positive or negative selection settings. A positive selection screen allows for the identification of genes whose knockout by sgRNAs gives a growth advantage to a cell in a complex population (e.g. survival or selectable phenotype). Genome-wide CRISPR-Cas9 screens in a setting of positive selection have discovered gene mutations that confer drug resistance, resistance to bacterial toxins and genes involved in metastasis [1, 4, 5, 7, 10]. For instance, using this approach Chen et al. discovered key genes involved in early, late and metastatic cancer [1]. More specifically, they identified tumor suppressor genes whose knockout by sgRNAs triggered cell proliferation and increased metastasis formation. Contrarily, a negative selection or *dropout* screen can identify genes whose knockout by sgRNAs cause the depletion of the cells. In a setting of negative selection one aims to identify oncogenic drivers, e.g. those genes that cause the formation, or supports the progression, of a cancer. While positive selection screens proved quite successful so far, initial negative selection screens by CRISPR-Cas9 detected many highly essential genes as screening hits [1, 3, 5–7]. These genes are required for the proliferation and survival of human cancer cell lines and include factors for RNA transcription and DNA replication [1, 3, 5–7]. These studies also found many previously uncharacterized genes involved in RNA processing demonstrating that CRISPR-Cas9 screens are a valid approach for the identification of genetic dependencies [3, 6]. In an attempt to identify new therapeutic targets, a recent negative selection study focused on a few hundred chromatin regulatory genes [11]. In this work the authors showed that CRISPR-Cas9 mutagenesis directed to exons encoding functionally important protein domains resulted in a higher efficiency [11]. Several genes were found to be indispensable for cell survival [11]. However, it is not known whether other important fitness genes can be identified besides the known oncogenes in EGFR and NRAS mutant cells in a whole-genome CRISPR-Cas9 negative selection screen.

Using a genome-wide sgRNA library in two human cancer cell lines with known mutations we show that CRISPR-Cas9 dropout screens can differentiate oncogenic drivers and pathways from the expected key survival genes. We exemplify this with the identification of EGFR as one of the top hits in the EGFR mutated HCC-827 line and NRAS and MAP2K1 (MEK1) among the top hits in the NRAS mutated CHP-212 line. In addition, we discover putative dependencies including TBK1 and TRIB2. Our data show that whole genome CRISPR dropout screens allow for the identification of oncogenic drivers as well as essential genes for survival that might be suitable for drug targeting.

## Results

### CRISPR-Cas9 screen and identification of essential genes involved in fundamental cellular processes

**Fig. 1:**
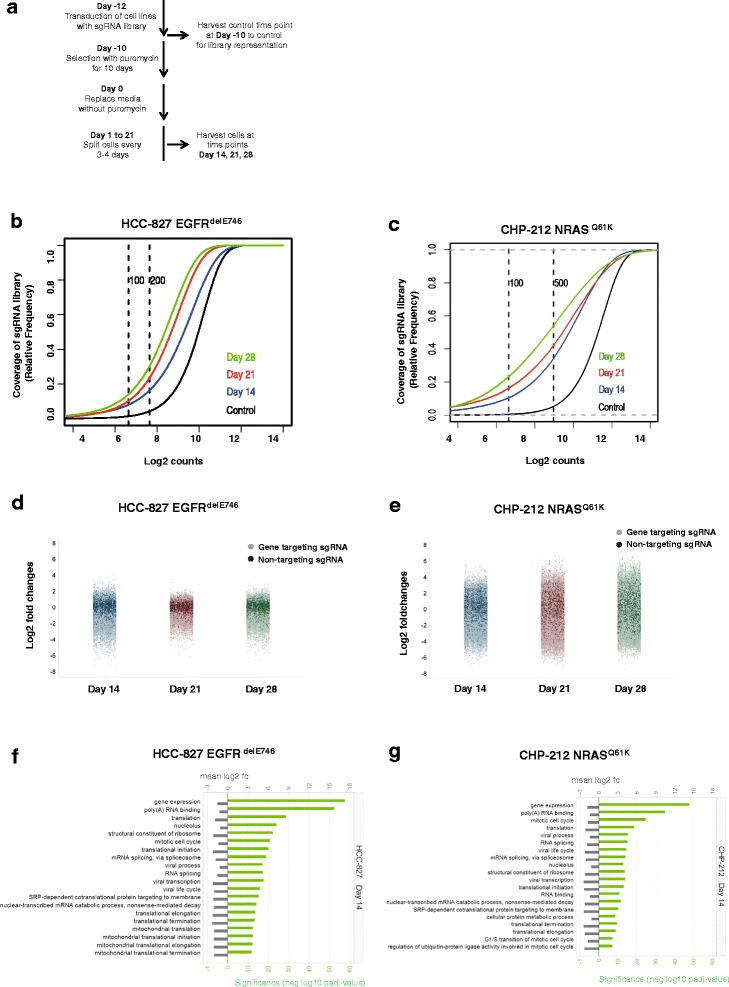
Representation of whole genome sgRNA library at different time points. **a** Schematic representation of the negative loss-of-function screen using lung cancer cell line HCC-827 and neuroblastoma cell line CHP-212. **b** Cumulative frequency of sgRNAs by deep sequencing at control time point (day −10), day 14, day 21, and day 28 for HCC-827 cell line. Shift in the curves at days 14, 21, and 28 represents the depletion of essential sgRNAs. Each time point was measured in duplicates. **c** Same as in **b**) but for CHP-212 cell line. **d** Plots of normalized sgRNA reads for HCC-827 cell line at time points day 14, day 21, and day 28. Dark colored dots represent the 1 000 non-targeting control sgRNAs and light colored dots represent the 57 096 targeting sgRNAs. Each time point was measured in duplicates and log2 of median fold changes versus the control time point (day −10) are represented. **e** Same as in **d**) but for CHP-212 cell line. **f** Gene ontology terms describing sgRNAs and genes whose knockdown causes under-representation of HCC-827 cells at day 14. **g** Gene ontology terms describing sgRNAs and genes whose knockdown cause under-representation of CHP-212 cells at day 14

To investigate whether pooled whole-genome CRISPR-Cas9 screening is an appropriate means to identify oncogenic drivers and novel dependencies we selected two human cancer cell lines with known mutations: (1) the neuroblastoma-derived cell line CHP-212, which carries a RAS (NRAS) Q61K mutation and is highly sensitive to MEK inhibitors [12, 13]; (2) the lung cancer cell line HCC-827, which carries a deletion in the epidermal growth factor receptor (EGFR) delE746 and is sensitive to EGFR inhibitors including Gefitinib and Erlotinib [14]. We introduced a human sgRNA library consisting of 57 096 unique sgRNAs (3 sgRNAs/gene) and 1 000 non-targeting control sgRNAs [5] into CHP-212 and HCC-827 cells by lentiviral transduction. Cells were then grown under puromycin selection for 10 days, and genomic DNA samples were collected at days 14, 21, and 28 thereafter without any selection pressure. Experiments were conducted in duplicates (Fig. 1a).

Using deep sequencing we found that the diversity of sgRNAs was reduced over time as expected (Fig. 1b, c, Additional file 1: Figure S1a, b). The shift between control and days 14, 21 and 28 indicates the specific depletion of sets of sgRNAs (Fig. 1b, c). Our data revealed high coverage of the sgRNA library: at the control time point 96.0 % of the sgRNAs were represented by at least 100 sequencing reads in HCC-827 and 98.9 % in CHP-212 cells, respectively (Additional file 1: Figure S1a, b). The percentage of sgRNAs detected significantly decreased after 14, 21, and 28 days as expected (Additional file 1: Figure S1a, b). The obtained sgRNA representations showed high concordance between technical replicates of CHP-212 cells (average correlation, ρ = 0.94) and HCC-827 cells (average correlation, ρ = 0.92) (Additional file 2: Table S1). High correlation was also found between the controls of HCC-827 and CHP-212 cells (average correlation, ρ = 0.93), thus indicating high reproducibility even between different biological infections (Additional file 3: Table S2). Next, we calculated log2 fold changes by taking the logarithm of sgRNA counts for one gene at each time point divided by the average of the sgRNA counts of that gene at the control time point (day −10). We found that the overall technical variability across the screen was low as the distribution of non-targeting versus targeting sgRNAs was consistent across all time points within each cell line (Fig. 1d, e). Variability was higher for the CHP-212 than for the HCC-827 cell line (Fig. 1d, e). Finally, principal components analysis (PCA) showed that replicates at control time points cluster together better than those at later time points (days 14, 21 and 28), thus suggesting that the variability between replicates increases with time as response to the depletion of the target genes (Additional file 1: Figure S2a, b). All together these data indicate robustness of our CRISPR-Cas9 screen based on high coverage of the sgRNA library, low technical variability for the same sgRNA across the different time points, and high reproducibility between replicates across the whole experiment. However, as previously described in [11, 15], we also observed variability between individual sgRNAs targeting different loci of the same gene (Additional file 1: Figure S3a, b). Thus, we decided first to conduct further analyses at sgRNA level rather than at gene level.

We analyzed the sgRNA distributions to assess whether perturbing gene function confers a growth advantage or disadvantage to the cells. Of the 57 096 unique sgRNAs used in the screen, we found a significant portion leading to under-representation of the cells at the different time points analyzed. Using gene set enrichment analysis (GSEA) [16], we identified that sgRNAs with decreased abundance in cells at day 14 target genes whose function is essential for cell survival such as those involved in fundamental cellular processes (e.g. cell cycle and mitosis, DNA replication, protein translation, RNA splicing, non-sense mediated decay, and RNA processing), key components of cellular organelles (e.g. ribosomal, mitochondrial and nucleolar proteins), as well as those encoding proteins with RNA and poly(A)-RNA binding functions (Fig. 1f, g). These results were confirmed at the later time points (data not shown). Thus, our analysis indicates that loss-of-function of these essential genes after 14 days of the screen results in growth disadvantage to the cells from both lineages.

### Oncogenic driver mutations can be identified by whole-genome CRISPR-Cas9 screen

**Fig. 2:**
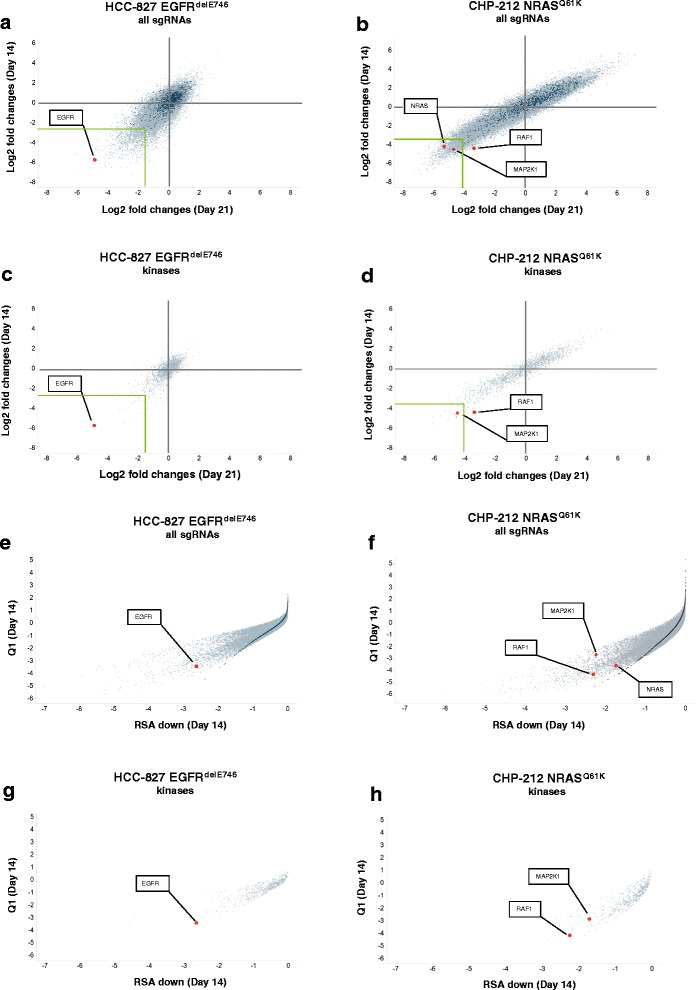
sgRNAs depleted in the whole genome screen. **a** Scatterplot representing fold changes of the 57 096 targeting sgRNAs in the HCC-827 cell line at day 14 and day 21. Fold changes at day 14 or day 21 were calculated compared to the control time point at day −10. All time points were measured in duplicates and median fold changes are shown. Dark green colored dots represent the 1 000 non-targeting control sgRNAs and grey colored dots represent the 57 096 targeting sgRNAs. Genes of interest were annotated by the software Spotfire and visualization was further enhanced by red colored dots. **b** Same as in **a**) but for CHP-212 cell line. **c** Scatterplot of fold changes of 1 571 kinases in the HCC-827 cell line at time points day 14 and day 21 versus control time point day −10. **d** Same as **c**) but for HCC-827 cells. **e**, **f** Scatterplots for Q1 and RSA down of 57 096 targeting sgRNAs in the HCC-827 (**e**) and CHP-212 cell lines (**f**) at time point day 14. Dark green colored dots represent the 1 000 non-targeting control sgRNAs. **g**, **h** Scatterplot for Q1 and RSA down of the 1 571 sgRNAs for kinases are shown for the HCC-827 (**e**) and CHP-212 (**f**) cell lines

To answer the question of whether a negative selection CRISPR-Cas9 screen can identify genes playing a non-redundant role in the oncogenic driver pathways, we deployed two different approaches: (1) we analyzed fold changes for all 57 096 sgRNAs; and (2) we focused on the 1 751 sgRNAs which target kinases. Fold changes for time points day 14, 21, and 28 were calculated as change in frequency of the respective sgRNA compared to the control time point at day −10. We compared fold changes of all sgRNAs from day 14 versus day 21 and found that most of the 1 000 non-targeting control sgRNAs overlaid with the majority of all targeting sgRNAs (Fig. 2a, b). This indicates that many data points fall into the background variability of the CRISPR screen (Fig. 2a, b). As a threshold level we used the fifth percentile of the fold changes of the depleted sgRNA (Fig. 2). This includes 1 450 sgRNAs of all genes from a total of 57 096 sgRNAs (Fig. 2a) and 22 sgRNAs from kinase sgRNAs (Fig. 2c) for the HCC-827 cell line. For the CHP-212 cell line, 1 462 sgRNAs are among the 5 % most depleted sgRNAs for all genes and 24 sgRNAs for kinases (Fig. 2b, d). We provide the lists of 1 000 most depleted genes for the HCC-827 and CHP-212 cell lines (Additional files 4 and 5: Tables S3 and S4). We found EGFR scoring high among the strongest depleted genes for the EGFR-mutant HCC-827 cell line being well above the threshold level (Fig. 2a). Similarly, NRAS and MAP2K1 – a kinase downstream of NRAS [17] - were among the most depleted genes for the NRAS-mutant cell line CHP-212 within the threshold level (Fig. 2b). RAF1, another kinase downstream of NRAS, was found to be below the threshold level (Fig. 2b). These data show that oncogenic drivers and pathways can be differentiated from the multitude of key survival genes in our CRISPR-Cas9 screen, indicating that an oncogenic driver mutation causes a strong dependency.

Then, we further examined the range of depleted genes focusing our analysis on kinases, depicting the fold changes of sgRNAs targeting kinases on days 14 and 21 (Fig. 2c, d). Again, we found EGFR and MAP2K1 among the most depleted of all kinases (Fig. 2c, d); these genes were identified at all time points, and confirmed by replicates from the same time point (Additional file 1: Figure S4a-f). Of note, sgRNAs for MAP2K1, NRAS and RAF1 were only depleted in CHP-212 cells but not in HCC-827 cells excluding unspecific effects of the respective sgRNAs (Additional file 1: Figure S4g). Similarly, EGFR was depleted in the HCC-827 cell line but not in the NRAS mutant CHP-212 cell line (Additional file 1: Figure S4h). For each cell line, we report the lists of the top 20 kinase-targeting sgRNAs with the strongest depletion at each of the three time points analyzed (Tables 1 and 2). EGFR was at the top position at all three time points in the EGFR-mutant HCC-827 line (Table 1). Similarly, MAP2K1 was among the top hits for the NRAS-mutant CHP-212 line (Table 2). RNA-sequencing of the parental HCC-827 and CHP-212 lines confirmed that our top candidate genes were highly expressed in the respective cells (Additional files 6 and 7: Tables S5 and S6). To determine potential off-target effects of the sgRNAs we compared depletion of sgRNAs to the expression of the respective genes. We found that less than 10 % of the top 5 % depleted genes are not expressed in the HCC-827 cell line and <12.5 % for the CHP-212 cell line (Figure S5a, b). This indicates that off-target effects of the sgRNA library are in a lower range and that 9 of 10 hits are likely valid candidates.

**Table 1:** List of top 20 sgRNAs targeting kinases for HCC-827 cells

Day 14			Day 21			Day 28		
Kinase	sgRNA	Log2 fold change	Kinase	sgRNA	Log2 fold change	Kinase	sgRNA	Log2 fold change
EGFR	HGLibA_14637	−5.61	EGFR	HGLibA_14637	−4.88	EGFR	HGLibA_14637	−5.49
NRBP1	HGLibA_39943	−5.37	CHEK1	HGLibA_09418	−4.81	NRBP1	HGLibA_39943	−5.37
TP53RK	HGLibA_58364	−5.08	LTK	HGLibA_34898	−3.67	PDPK1	HGLibA_43219	−4.54
RIPK2	HGLibA_48607	−4.64	SRPK2	HGLibA_54308	−3.45	CDK11A	HGLibA_08724	−3.98
CHEK1	HGLibA_09418	−4.58	CDK11A	HGLibA_08724	−3.39	TBK1	HGLibA_55891	−3.78
PIK3R3	HGLibA_43942	−4.54	WEE1	HGLibA_61525	−3.22	CHEK1	HGLibA_09418	−3.57
WEE1	HGLibA_61525	−4.10	RET	HGLibA_48160	−3.17	WEE1	HGLibA_61525	−3.48
TBK1	HGLibA_55891	−4.06	TBK1	HGLibA_55891	−3.00	CDK2	HGLibA_08756	−3.37
DYRK2	HGLibA_14277	−3.96	NRBP1	HGLibA_39943	−2.88	TP53RK	HGLibA_58364	−2.97
MERTK	HGLibA_36194	−3.78	PHKG1	HGLibA_43699	−2.64	TLK1	HGLibA_56909	−2.82
CLK2	HGLibA_10058	−3.49	TP53RK	HGLibA_58364	−2.48	RET	HGLibA_48160	−2.81
PKMYT1	HGLibA_44123	−3.41	PDPK1	HGLibA_43219	−2.47	PKMYT1	HGLibA_44123	−2.80
CDK11A	HGLibA_08726	−3.40	PRKAA1	HGLibA_45780	−2.43	MUSK	HGLibA_37865	−2.73
PDPK1	HGLibA_43219	−3.36	SCYL1	HGLibA_50372	−2.32	PHKG1	HGLibA_43699	−2.72
IKBKE	HGLibA_30211	−3.30	PKMYT1	HGLibA_44123	−2.31	PIK3R3	HGLibA_43942	−2.67
CSNK1D	HGLibA_11388	−3.28	PRKCD	HGLibA_45826	−2.29	DYRK2	HGLibA_14277	−2.62
CDK11B	HGLibA_08729	−3.21	TBK1	HGLibA_55890	−2.27	TBK1	HGLibA_55889	−2.52
CDK4	HGLibA_08771	−3.17	CDK12	HGLibA_08731	−2.25	PAK4	HGLibA_42338	−2.46
AURKA	HGLibA_03873	−3.14	CLK2	HGLibA_10058	−2.25	RIPK2	HGLibA_48607	−2.44
LTK	HGLibA_34900	−3.02	CDK2	HGLibA_08756	−2.22	PRKAA1	HGLibA_45780	−2.43

**Table 2:** List of top 20 sgRNAs targeting kinases for CHP-212 cells

Day 14			Day 21			Day 28		
Kinase	sgRNA	Log2 fold change	Kinase	sgRNA	Log2 fold change	Kinase	sgRNA	Log2 fold change
TRIB2	HGLibA_58681	−5.96	STRADA	HGLibA_54860	−6.17	CLK2	HGLibA_10058	−5.49
STRADA	HGLibA_54860	−5.41	CDC7	HGLibA_08595	−6.09	CHEK1	HGLibA_09420	−5.37
BRD2	HGLibA_04772	−5.31	TRIB2	HGLibA_58681	−6.06	RIOK2	HGLibA_48597	−4.54
TEX14	HGLibA_56351	−5.14	PDK1	HGLibA_43184	−5.65	MAP2K1	HGLibA_35426	−3.98
MAP2K6	HGLibA_35443	−4.83	VRK1	HGLibA_61113	−5.64	GAK	HGLibA_18491	−3.78
CDC7	HGLibA_08595	−4.77	RIOK2	HGLibA_48597	−5.27	AATK	HGLibA_00089	−3.57
VRK1	HGLibA_61113	−4.75	BRD2	HGLibA_04772	−5.21	ACTR2	HGLibA_00655	−3.48
PDK1	HGLibA_43184	−4.59	TEX14	HGLibA_56351	−5.14	TP53RK	HGLibA_58364	−3.37
RPS6KB1	HGLibA_49465	−4.58	YES1	HGLibA_61950	−5.11	BRD2	HGLibA_04772	−2.97
ZAP70	HGLibA_62062	−4.57	GAK	HGLibA_18491	−5.02	PDK1	HGLibA_43184	−2.82
MAP2K1	HGLibA_35426	−4.52	PTK2	HGLibA_46667	−4.89	BRD2	HGLibA_04773	−2.81
RAF1	HGLibA_47416	−4.37	CHEK1	HGLibA_09418	−4.88	TRIB2	HGLibA_58681	−2.80
KIT	HGLibA_32303	−4.36	RIPK1	HGLibA_48603	−4.88	YES1	HGLibA_61950	−2.73
RIOK2	HGLibA_48597	−4.35	CHEK1	HGLibA_09420	−4.86	CDC7	HGLibA_08595	−2.72
SBK2	HGLibA_50091	−4.33	ZAP70	HGLibA_62062	−4.71	VRK1	HGLibA_61113	−2.67
GAK	HGLibA_18491	−4.32	RPS6KB1	HGLibA_49465	−4.70	STK38L	HGLibA_54791	−2.62
SYK	HGLibA_55237	−4.21	MAP2K1	HGLibA_35426	−4.60	SBK2	HGLibA_50091	−2.52
PTK6	HGLibA_46673	−4.11	MAP2K6	HGLibA_35443	−4.59	PKMYT1	HGLibA_44123	−2.46
RIOK1	HGLibA_48595	−3.98	KIT	HGLibA_32303	−4.57	NEK1	HGLibA_38926	−2.44
IGF1R	HGLibA_30081	−3.92	RPS6KA4	HGLibA_49455	−4.53	CDK11A	HGLibA_08726	−2.43

We observed that while some sgRNAs for a given gene had a very strong effect on depletion others were mostly ineffective (Figure S4g, h). This low sgRNA efficiency has been also described by other studies and could be due to several factors including the low expression of Cas9, the non-optimal sequence of the sgRNA, a low chromatin accessibility, and ultimately an insufficient editing of the locus [15]. Thus, we decided to add another analytical approach which encompasses all 3 sgRNAs. To compute a gene-based hit calling, we applied a redundant siRNA activity (RSA) statistics [18] on the fold changes obtained at day 14 versus the control time point (day −10). Our results confirmed the previously identified hits, EGFR, RAF1 and MAP2K1 among the very top hits in the plots for all sgRNAs and for kinase sgRNAs only (Fig. 2e-h). Taken together, we conclude that single sgRNA analysis and gene based analysis of all 3 sgRNAs yield a comparable outcome.

### Drug sensitivity to specific kinase inhibitors correlates with sgRNA depletion of respective kinases

**Fig. 3:**
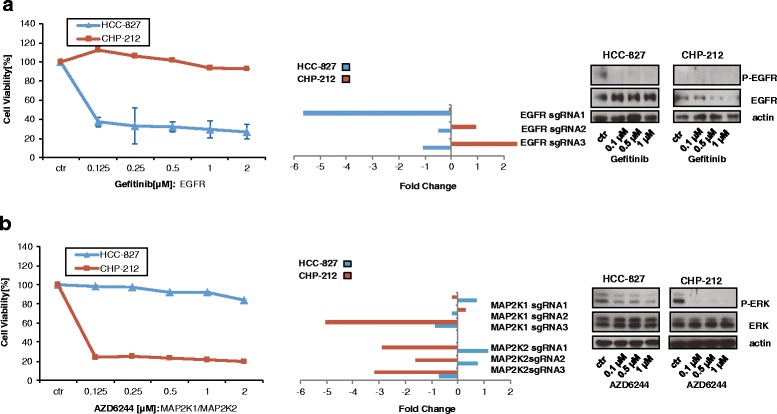
Depletion of kinases EGFR and MAP2K1 correlates with sensitivity towards EGFR and MEK inhibitors. **a** *left panel*: HCC-827 and CHP-212 cells were treated with indicated concentrations of Gefitinib for 72 h. Then, cell viability was measured by Cell Titer Glo according to the manufacturer’s instructions. *Middle panel*: Fold change for the three independent sgRNAs for EGFR from the screen are depicted at time point day 14. *Right panel*: HCC-827 and CHP-212 cells were treated for 2 h with the indicated concentrations of Gefitinib. Then, cells were lysed and analysed by Western blot. **b** same as **a**) but the MEK inhibitor AZD6244 was used instead (*left panel*) and fold change for sgRNAs for MAP2K1 and MAP2K3 from the screen are depicted (*middle panel*). *Right panel*: HCC-827 and CHP-212 cells were treated for 2 h with the indicated concentrations of AZD6244. Then, cells were lysed and analysed by Western blot

To validate the findings of the CRISPR-Cas9 screen, we investigated the correlation between the sgRNA fold depletion and the drug sensitivity of the cell lines. The EGFR-mutant HCC-827 cell line was completely refractory towards MEK inhibition but highly sensitive to the EGFR inhibitor Gefitinib [13, 14] (Fig. 3a, left panel). In contrast, the NRAS-mutant cell line CHP-212 was highly sensitive towards MEK inhibition but insensitive towards Gefitinib (Fig. 3b, left panel). As expected, the drug sensitivity of the two cell lines broadly agreed with the sgRNA-based depletion of these genes observed in the screen (Fig. 3a, b, middle panel). We found that the fold changes of sgRNAs strongly correlated with sensitivity towards respective inhibitors of the target (correlation ρ = 0,99). These results were corroborated by the other MEK inhibitor MEK162 and the EGFR inhibitor Erlotinib (Additional file 1: Figure S6). In conclusion, our data show that sgRNA depletion strongly correlates with sensitivity to respective kinase inhibitors of the associated pathways in cell viability assays.

**Fig. 4:**
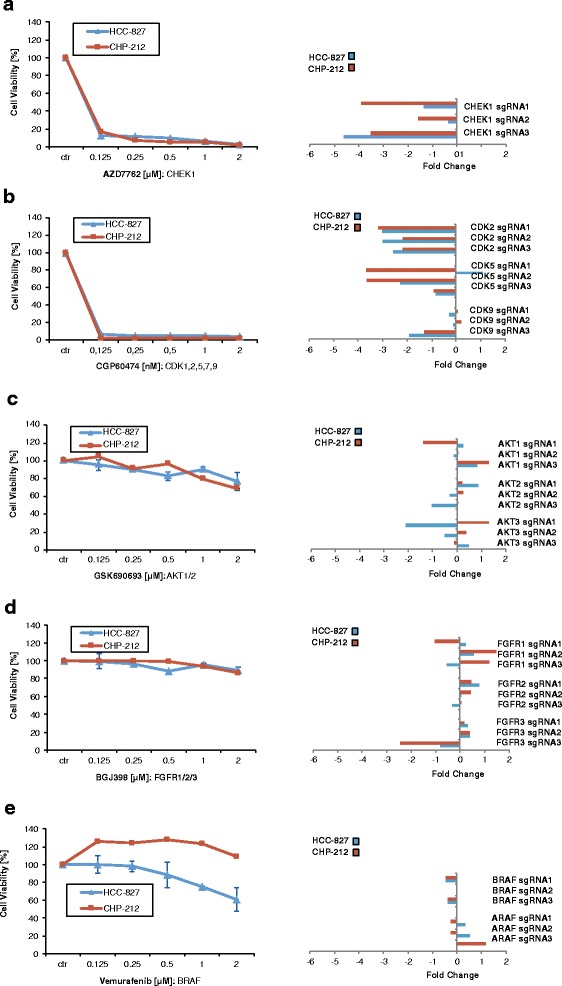
Validation of target kinases by inhibitors. **a** left panel: HCC-827 and CHP-212 cells were treated with indicated concentrations of CHEK1 inhibitor AZD7762 for 72 h. Then, cell viability was measured by Cell Titer Glo according to the manufacturer’s instructions. *Middle panel*: Fold changes for three independent sgRNAs for CHEK1 inhibitor AZD7762 are depicted at time point day 14. **b** Same as **a**) but CDK1,2,5,7,9 inhibitor and respective sgRNAs are shown. **c** Same as **a**) but AKT1/2/inhibitor MK2206 and respective sgRNAs are shown. **d** Same as **a**) but FGFR inhibitor BGJ398 and respective sgRNAs are shown. **e** Same as **a**) but BRAF inhibitor Vemurafenib and respective sgRNAs are shown

In another approach, we wanted to validate novel hits from the candidate list for which kinase inhibitors are available. For instance CHEK1 was a hit in both cell lines (Tables 1 and 2). Thus, we were curious to investigate the effect of the CHEK1 inhibitor AZD7762 on cell viability. Indeed, the CHEK1 inhibitor AZD7762 strongly blocked cell viability in both cell lines with EC50 values for CHP-212 and HCC-827, respectively (Fig. 4a). In addition, members of the cyclin dependent kinase (CDK) family were also abundant among the candidate list of our screen in both cell lines represented by depletion of CDK2, CDK4, CLK2, CDC7 and CDK11A,B (Tables 1 and 2). Indeed, depletion of the CDK2 and CDK5 correlated with sensitivity towards the CDK inhibitor CGP60474 for HCC-827 cells and for CHP-212 cells (Fig. 4b). Of note, for both CHEK1 and CDK2/5 most sgRNAs resulted in a strong depletion of genes from the screen (Fig. 4a, b: middle panels). As a control, we thought to determine kinases whose sgRNA counts were found to be stable or increased throughout the screen (Fig. 4c-e: middle panel) and for which kinase inhibitors are available. We found that sgRNAs for AKT1/2/3, FGFR1/2/3, and A- or BRAF inhibitors did not change significantly throughout the screen in both cell lines (Fig. 4c-e, right panel). As expected, inhibitors against these kinases had no effect on cell viability in both cell lines (Fig. 4c-e, left panel). Taken together, these data show that depletion of sgRNAs indicated sensitivity towards inhibitors of the respective target genes and that cell lines are highly sensitive to inhibitors of top ranking kinases from our screen.

### Identification of putative novel dependencies TBK1 and TRIB2

**Fig. 5:**
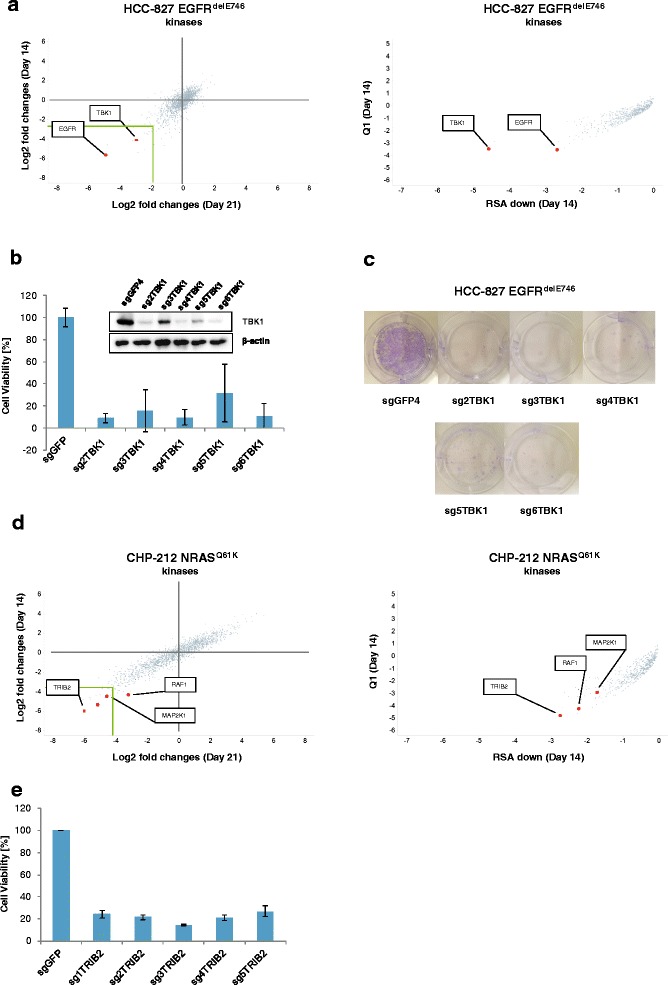
Validation of the screen by knock-out of TBK1 and TRIB2. **a** *Left side*: Scatterplot of fold changes for the 1 462 kinase sgRNAs of the HCC-827 cell line at time point day 14 and time point day 21 versus control time point day −10. sgRNAs against TBK1 were annotated by the software Spotfire and visualization was further enhanced by red colored dots. *Rigth side*: Scatterplot of Q1 and RSA down for the 1 462 kinase sgRNAs of the HCC-827 cell line at time point day 14 versus control time point day −10. **b** HCC-827 cell line was transduced with a non-targeting sgRNA against GFP and 5 different sgRNAs against TBK1 (TBK1_2 is a sgRNA included in the genome-wide screens while the other 4 were newly designed for this validation). Cell viability was measured 18days after viral transduction. **c** Equal number of cells transduced with a non-targeting sgRNA against GFP and 5 different sgRNAs against TBK1. Crystal violet staining was performed after 28 days. **d**-**e** Same as in a-c but CHP212 cells were used with the candidate TRIB2

Finally, we wanted to describe unexpected dependencies in these cell lines. The tank-binding kinase TBK1 was among the strongest hits identified by the two different analysis approaches in the HCC-827 cell line (Fig. 5a, b). TBK1 was at position 5 in the sgRNA-based analysis and at position 1 in the gene-based analysis (Fig. 5a). TBK1 was described as co-synthetic lethal in KRAS mutant lung cancer [19] but, so far, it has not been associated with EGFR mutant cancer. We found that sgRNAs targeting TBK1 decrease cell viability for HCC-827 (Fig. 5b). Efficacy for knock-out of different sgRNA correlated with the degree of decrease of cell viability (Fig. 5b). In addition, in a colony formation assay, knock-down of TBK1 by different sgRNAs significantly reduced ability of cells to form colonies (Fig. 5c). TBK1 shows a strong expression in the HCC-827 cell line similarly to EGFR (Additional file 6: Table S5). We observed that many potential hits are strongly expressed (Additional file 6: Table S5). Two recent studies observed that CRISPR/Cas9 screens may generate false-positive hits for genes with high copy numbers or genes in amplified regions [20, 21]. The authors describe that DNA breaks by CRISPR/Cas9 in amplified regions cause an antiproliferative effect independent of the respective gene targeted by the sgRNA [20]. Using the canSAR database [22], we indeed found that TBK1 has 10 copy number variants in the parental HCC-827 cell line. While further studies are needed to prove that TBK1 is a true dependency, we can conclude that these experimental data validate the findings of our CRISPR screen.

In addition, we validated another hit for the CHP-212 cell line. We found that tribbles pseudokinase 2 (TRIB2) was scoring high in both analyses (Fig. 5d). 5 different sgRNAs, 4 of which newly designed for the targeted validation, reduced cell viability compared to a non-targeting control for TRIB2 (Fig. 5e). Again, these results demonstrate the high reproducibility of the screen. A search in the canSAR database [22] showed that TRIB2 has 14 different copies in the CHP-212 cell line and we conclude that further studies are needed to validate TRIB2. The role of TRIB2 in cancer and its potential druggability merit further investigations.

## Discussion

Here, we found that screening with a commercially available CRISPR-Cas9 library enables the identification of oncogenic driver mutations and the discovery of novel putative therapeutic targets. We show that relevant druggable targets can be identified through a whole-genome approach with known massive depletion of genes involved in basic cellular functions including cell transcription and replication. We chose two human cancer cell lines with known mutations: EGFR mutations are found in 10–15 % of lung cancer patients. NRAS mutations activate downstream kinases including RAF and MEK1/2 (MAP2K1/2) [17], and preclinical and early clinical data showed that NRAS-mutant cell lines and tumors of cancer patients are highly sensitive to MEK1/2 inhibitors [13, 23]. SgRNAs targeting the two driver mutations, used as intrinsic controls for our screen, showed up in the top-candidate list. SgRNAs targeting the driver mutations could be identified at days 14, 21 and 28 indicating that knock-out efficiency at day 14 suffices. We noticed that EGFR ranked higher for the HCC-827 cell line than MAP2K1 for the CHP-212 cell line. This is consistent with the fact that we found more background noise in the CHP-212 line as indicated by the higher variability of non-targeting controls. Whether this is specific to CHP-212 cells or might occur also in other cell lines has to be investigated by further CRISPR-Cas9 screens in more cell lines. Nevertheless, focusing the analysis on kinases revealed the repetitive appearance of MAP2K1 and RAF1 among the most depleted kinases at most time points (Table 2). This indicates that the hyperactivity of the RAS pathway due to the NRAS mutations is presented by the identification of the respective downstream kinases.

Further, we found that the expected hits from the CRISPR screen are strongly expressed (Fig. 2, Additional file 1: Figure S5). This is interesting, since RNA sequencing and expression may help to identify genes which are not expressed and therefore delineate potential CRISPR off-target effects. In our data we estimated the sgRNA off-target effect to be below 10 % and 12.5 % for HCC-827 cells and CHP-212 cells, respectively. This would imply that at least 8 or 9 out of 10 hits could be true hits that may be valid for further investigations.

Besides the expected targets EGFR and MAP2K1, our screen revealed also other putative drug targets that showed strong depletion. For example, we found TBK1, a kinase so far associated with KRAS mutations, as a top hit in HCC-827 cells (Fig. 5). TBK1 was shown to be co-lethal in KRAS mutant lung cancer and it has been investigated as a potential target in triple-negative breast cancer [19, 24]. Whether TBK1 plays a role in EGFR mutant lung cancer is currently not known. For CHP-212 we were able to validate the screening results for the cell cycle kinase TRIB2 (Fig. 5). We took advantage of the three recent studies published [3, 6, 25] to check whether the genes described in our work were reported to be “essential genes” in previous screens (Additional file 1: Figures S3 and S4). We can confirm that TBK1 and TRIB2 are not present in any of the three published lists of essential genes [3]. The finding of these kinases is highly interesting and especially their role in cancer is poorly understood. However, it has to be taken into consideration that CRISPR-Cas9 may generate false-positive hits since genes with high copy numbers or areas with amplified regions are especially sensitive to DSB by Cas9 [20, 21]. Thus, identified potential hits need further confirmation by shRNA experiments and studies on more cell lines. The hypothesis that these kinases might be suitable co-drug targets for treatment of cancer patients merits further investigations.

Currently, it is speculated whether the diploid or polyploid genome of mammalian cells would be an obstacle for whole-genome CRISPR-Cas9 screening [26]. The cell line CHP-212 was found to be diploid whereas the HCC-827 cells are tri- to hexaploid (Additional file 1: Figure S7). This indicates that chromosomal aberrations are not necessarily a barrier for the identification of driver mutations.

The outcome and strength of CRISPR-Cas9-mediated genetic screening in general, and for loss-of-function dropout screen in particular, is dependent on the efficiency of gene knockout. Gene knockout efficiency is influenced by the expression levels of Cas9, the sequence of the sgRNA [11], and the chromosomal context [11]. Recently, it was found that knock-out of genes is more efficient if Cas9 is directed to highly conserved, functional domains [11]. In this work we confirm that sgRNAs targeting different exons of the same gene can lead to different efficiency in the phenotype. This outcome confirms previous data showing that independent sgRNAs can give high variability in the phenotype penetrance [1, 10, 11]. We also observed high variability of sgRNAs targeting the same gene. For example, one sgRNA targeting EGFR scored extremely high while the two others remain ineffective (Additional file 1: Figure S4g). The reason for the different sgRNA efficacy could not be studied further here. However, we could show that both analytic approaches, e.g. 1) focusing on fold change of single sgRNAs and 2) combining all 3 sgRNAs per gene, yield reliable results (Fig. 2). However, we conclude that analyzing individual sgRNAs is superior and more reliable since EGFR - a well-studied and defined oncogene which is highly relevant as a predictive marker for treatment of lung cancer patients - showed a score higher in single sgRNA analysis than in the combined sgRNA setting (Fig. 2). In conclusion, screening with more sgRNAs per gene might increase robustness of the screening. Thus, 2nd and 3rd generation CRISPR/Cas9 sgRNA libraries designed with novel prediction algorithms might overcome these limitations. Further, the integration of hits generated from CRISPR screens in other cancer cell lines might help filter out pan-lethal targets and highlight important unknown dependencies. In fact if we disregard from our analysis those genes found to be essential in recent papers [3, 6] and those that score in both our cell lines, EGFR and NRAS rank respectively 2nd and 7th based on their cumulative screening score (e.g. the cumulative log fold changes at all three time points) and their expression level (Additional files 8 and 9: Tables S7 and S8).

For our screen, we have observed that 3 independent sgRNAs were sufficient to identify known oncogenes and novel dependencies. However, we cannot exclude that targets were missed because all 3 sgRNAs for a single gene were ineffective in inducing a phenotypically relevant knockout.

## Conclusions

In summary, we found that oncogenic driver mutations and target kinases can be identified by a whole-genome CRISPR-Cas9 dropout screen. CRISPR-Cas9 screening supports the discovery of novel drug targets.

## Methods

### Chemicals

AZD6244, MEK162 and Erlotinib were purchased from Selleck Chemical. Gefitinib were kindly provided by Viktoras Frismantas and Jean-Pierre Bourquin, Childrens University Hospital Zurich. All inhibitors were solubilized in dimethyl sulfoxide (DMSO) at stock concentrations of 10 mM.

### Cell culture

CHP-212 and HCC-827 cells were purchased from ATCC. CHP-212 cells were cultured in DMEM and F12 medium (1:1) supplemented with 10 % fetal calf serum (FCS) and 1 mM L-glutamine. HCC-827 cells were cultured in RPMI-1640 supplemented with 10 % FCS.

### Karyotyping of cell lines

A T25 cell culture flask with 5 ml media and approximately 60 % confluence was obtained for each sample. Cultures were incubated with 25 μl colcemid (KaryoMAX Colcemid Solution, 10 μg/μl; Life Technologies, Zug, Switzerland) at 37 °C and 5 % CO2 for 24 h before harvest. Chromosome preparation was done using standard techniques [27]. Briefly, the adherent cells were detached using incubation with trypsin, followed by hypotonic treatment, fixation with methanol-acetic acid, slide preparation and GTG banding. Metaphases were searched and captured using an automated microscope equipped with a scanning software (Metafer, MetaSystems GmbH, Altlussheim, Germany). Metaphases were analyzed using the Metasystems IKAROS software.

### Lentivirus production and purification

GeCKO Library A was purchased from Addgene and library was purified according to manufacturer’s instructions. For lentivirus production, 30 T-75 flasks with HEK293T cells were seeded to reach 70–80 % confluence the day of transfection in DMEM supplemented with 10 % fetal bovine serum. 30 min prior to transfection, media was removed and fresh prewarmed media with 25 μM chloroquine was added. Transfection was performed using the CaCl2 method (https://web.stanford.edu/group/nolan). 20 ug of lentiCRISPR plasmid library, 10 ug of pVSVg, and 15 ug of psPAX2 (Addgene) were mixed with 61ul of 2M CaCl2, then 459ul of ddH2O was added, finally 500ul of 2xHBS (Na2HPO4 dibasic (5.25 g in 500 ml of water), 8.0 g NaCl 6.5 g HEPES (sodium salt) 10 ml Na2HPO4 stock solution, pH to 7.0 using NaOH or HCl) were added. Then, 2 ml of the DNA-Ca precipitate are added to each flask. After 12 h, the media was removed and fresh DMEM +10 % FCS media was added. After 48–72 h, the media was pooled and centrifuged at 3,000 rpm at 4 °C for 5 min twice to pellet cell debris. Then, the supernatant was ultracentrifuged at 25,000 rpm for 2 h at 4 °C (Sorvall) and then suspended overnight at 4 °C in 1 ml OPTI-MEM. Aliquots were stored at −80 °C.

### Large scale spin transduction

Cells were transduced virally with the sgRNA library by spinfection. Multiplicity of infection (MOI) was determined prior by infecting target cells with 1, 5, 10, 25, 50 μl of ultracentrifuged viral supernatant and selection with puromycin 48 h after viral transduction (the optimal puromycin concentrations were analyzed separately, 1ug/μl was found to kill 100 % of CHP-212 whereas HCC-827 required 4ug/μl), then viability was measured by cell titer glo 96 h after addition of puromycin. We aimed to cover each sgRNA by at least 100 reads for the control time point, resulting in 100 × 57 096 sgRNAs (=5.71 × 10^6^ cells). Assuming a MOI of 0.5 to ensure proper representation of the library and considering that 50 % are taken out of the pool at day 3 at least 4 × 5.71 × 10^6^ would be required. Finally, large-scale spinfection of 30 × 10^6^ cells (CHP-212 and HCC-827) was carried out with calculated volume of purified virus according to MOI of 0.5 in 24-well plates which resulted in coverage of 100 reads for >96 % of all sgRNAs and 200 reads for >95 % of all sgRNAs in both cell lines for the control time point (Fig. 1b, c). Standard media for each cell line (see Cell culture) for spinfection was supplemented with 8 ug/ml polybrene (Sigma). The 24-well plate was centrifuged at 2,250 rpm for 2 h at 24 °C. Immediately after the spin, old media was carefully aspirated and fresh media without polybrene was added to the cells.

### Depletion screen

On day 3, 50 % of cells were harvested for the baseline time point (control time point). Then, 4 μg/ul or 1 μg/μl of puromycin (0.5 μg/mL) was added to HCC-827 and CHP-212 cells for 10 days, respectively. On day 10, media was removed and fresh media without puromycin added. HCC-827 and CHP-212 were split every 3–4 days once confluence reached 70 - 80 %. After 14, 21, and 28 days, at least 30 × 10^6^ cells were harvested in duplicates for genomic DNA extraction and analysis.

### Genomic DNA sequencing

Extraction of DNA and further sample preparation was done as described previously by Shalem O. et al. [5]. Harvested cell pellets were thawed and genomic DNA was isolated with a Blood & Cell Culture Maxi kit (Qiagen). The PCRs for amplification of the library were performed in two steps [5]. First, sgRNAs were amplified from genomic DNA. According to [5], an input amount of 130 ug genomic DNA was used for 12 separate PCR reactions in 100ul using Herculase II Fusion DNA Polymerase (Agilent) to achieve 300X coverage over the sgRNA library (assuming 6.6 ug of gDNA for 10 [6] cells). The 12 generated PCR amplicons were then pooled. Amplification was carried out with 18 cycles for the first PCR. Primer sequences to amplify sgRNAs from genomic DNA are:

- F1 AATGGACTATCATATGCTTACCGTAACTTGAAAGTATTTCG
- R1 CTTTAGTTTGTATGTCTGTTGCTATTATGTCTACTATTCTTTCC

Second, another PCR was performed to attach Illumina adaptors and to barcode samples. This second PCR was done using 5ul of the product from the first PCR in 10 replicates in a reaction volume of 100 ul [5]. Primers for the second PCR contained in the forward primer a staggered length sequence to increase complexity of the library and an 8 bp barcode for multiplexing the different biological samples:

- F2 AATGATACGGCGACCACCGAGATCTACACTCTTTCCCTACACGACGCTCTTCCGATCT
- (1–9 bp variable length sequence) (8 bp barcode)tcttgtggaaaggacgaaacaccg
- R2 CAAGCAGAAGACGGCATACGAGATGTGACTGGAGTTCAGACGTGTGCTCTTCCGATCT
- tctactattctttcccctgcactgt

Amplification for the second PCR was done with 16 cycles (HCC-827) and 20 cycles (CHP-212). The amplicons from the second PCR were pooled and gel extracted. Sequencing was done with a HiSeq 2500 (Illumina). The raw sequencing reads are available in the NCBI Short Read Archive under the accession number SRP062971.

### Data processing and primary analysis

The reads of the raw FASTQ files were aligned against the FASTA file containing the designed sgRNA sequences from the library using the local alignment mode of the Bowtie2 aligner [28] allowing for one mismatch. After alignment, the number of aligned reads for each sgRNA sequence was calculated and normalized to the 75 percentile of the mean of the samples in the data set [29]. In order to deal with zero count sgRNAs in the computation of fold changes, a pseudo-count of 0.1 CPM was added to the normalized counts. After averaging the sgRNA values over the two replicates for each time point, we computed the log2 fold changes by dividing by the average of the baseline samples at the control time point (day −10) and then taking the base 2 logarithm. We calculated a cut-off for potential hits by considering only the 5 % most depleted sgRNAs from all depleted sgRNAs in a cell line. For the cell line HCC-827 at time point day 14, the 5 % threshold includes 1 450 sgRNAs for all genes or 22 sgRNAs for kinases from a total of 57 096 sgRNAs. This equals a fold change of < −2,66 for time point 14 and of < −1,8 for time point day 21. For CHP-212 cells, we calculated a cut-off at −3,6 for time point day 14 and −4,15 for time point day 21 accordingly. This includes 1 462 sgRNAs for all genes and 24 sgRNAs for kinases for the CHP-212 cell line at time point day 14.

Kinases in our screen were identified by using previously published data of all putative kinases found in the human genome [30]. Ranking of kinases was done according to calculated log2 fold changes. We also computed RSA scores as previously described in [18] using the log2 fold changes obtained at day 14 versus the control time point (day −10), as well as the Q1 (1st quartile) scores which are given as the mean of the lowest and second lowest sgRNA value of the three sgRNA values for a gene. Candidate genes were obtained by ranking their RSA-down score in ascending order.

### Gene set enrichment analysis

Gene set enrichment analyses were run using a custom Python script that performs standard one-tailed Kolmogorov-Smirnov tests [16] on the log2 fold ratios between the first time point T1 (Day 14) and the control T0 (Day −10). For each gene and each time point, we chose the log2 fold ratio that is maximal in terms of absolute values for sgRNAs targeting the same gene. As gene sets we used GO Biological Processes, GO Molecular Functions as well as GO Cellular Components and allowed for gene set sizes between 20 and 2000. Altogether we tested 1747 gene sets. We ranked the gene sets according to the significance values (negative log10 p-values) of the two one-tailed Kolmogorov-Smirnov tests for positive and negative fold changes and selected the top 20 gene sets (if any) above a threshold of 7 for the negative log10 p-value. p-values were multi-experiment adjusted using the Benjamini-Hochberg method. In order to assess the robustness of our analysis, we performed additional enrichment analysis with different test statistics and multi-experiment corrections and obtained very similar results (not shown).

### RNA sequencing

Total RNA was isolated in triplicates from CHP-212 and HCC-827 cells using the RNeasy Mini isolation kit including on-column DNase digestion according to the manufacturer’s instructions (Qiagen). RNA quality was assessed with the RNA 6000 Nano Kit (Agilent). RNA-seq libraries were prepared using the TruSeq Stranded mRNA Sample Prep kit v2 (Illumina) and sequenced in strand specific paired-end mode, 2x76bp, using the HiSeq2500 platform. Read quality was assessed by running FastQC (version 0.10) on the FASTQ files. Sequencing reads showed high quality, with a mean Phred score higher than 30 for all base positions. A total of 453 billion 76-base-pair (bp) paired-end reads was mapped to the human reference genome (hg38) and the human gene transcripts from Ensembl v76 [31] by using an in-house gene quantification pipeline [32]. On average, more than 97 % of the total reads were mapped to the genome or the transcripts, and more than 91 % to the exons and junctions (expressed reads). Genome and transcript alignments were used to calculate gene counts based on Ensembl gene IDs. The raw RNA-sequencing reads are available in the NCBI Short Read Archive under the accession number SRP062973.

### Western blot analysis

A total of 5 × 10^5^ cells were lysed for 30 min in ice-cold MPERM buffer supplemented with 25 mM NaF, 1 mM dithiothreitol, and complete protease inhibitor cocktail from Roche Diagnostics. Equal amounts of protein were separated by sodium dodecyl sulfate–polyacrylamide gel electrophoresis. Then, separated proteins were blotted onto a nitrocellulose membrane (GE Healthcare) followed by blocking with 5 % bovine serum albumin in phosphate-buffered saline/Tween (0.05 % Tween-20 in phosphate-buffered saline). The following antibodies were used: anti-phospho-ERK (P-p44/p42 (Tyr202/204, #9101, Cell Signaling Technology), anti-ERK (p44/p42, # 4695, Cell Signaling Technology), anti-phospho MEK (Ser298, #9128, Cell Signaling Technology), anti-MEK (# 8727, Cell Signaling Technology), anti-TBK1 (Cell Signaling Technology), and anti–tubulin (Sigma-Aldrich).

### Cell proliferation and viability assays

Cell proliferation was measured with the Cell-Titer-Glo Reagent (Promega) according to manufacturer’s instructions. Cells were plated in clear-bottomed 96-well plates at a density of 500–2500 cells per well. The next day, drugs were added at indicated concentrations and cell proliferation was measured 4 days later. Proliferation measurements were made using a standard 96-well plate luminometer/plate reader (Synergy 2, Biotek). Data are shown as relative values in which the luminescence at a given drug concentration is compared with that of untreated cells of the same type. All experimental points were set up in duplicate and were conducted at least 3 independent times. IC50 were calculated with GraphPad Prims. Correlation of fold changes of sgRNA downregulation and effect of drug treatment was calculated comparing the fold changes in sgRNAs with decrease in cell viability after drug treatment in Excel.

### Cloning of individual sgRNAs

sgRNAs for TBK1 and TRIB2 were designed by the sgRNA designer from the Broad Institute (http://www.broadinstitute.org/rnai/public/analysis-tools/sgrna-design). sgRNAs were then cloned into target vector lentiCRISPR_v2 (Addgene, Plasmid #52961) according to manufacturer’s instructions. sgRNAs for TBK1 include: TBK1_2 also used in the screen (CATAAGCTTCCTTCGTCCAG); TBK1_3 (CCTGAGTCTCGAGGAGGCCG); TBK1_4 (TCCACGTTATGATTTAGACG); TBK1_5 (ACATTTCCCTAAAACTACTG); TBK1_6 (GACAGCAGATTATCTCCAGG). sgRNAs for TRIB2 include: TRIB2_1 also used in the screen (AGAGTTTCAGCCCGAACCT); TRIB2_2 (TTAACTGAGCTCATGCCCCA); TRIB2_3 (CTATTAATACCGCCTCGCCG); TRIB2_4 (CCTTGTCTCCTGGTTACGAA); TRIB2_5 (AGCCTGTGCTGACCTCCGCG); TRIB2_6 (GCTGCCCTATTCACTTCTAA). Viruses were generated and transduced as described above. Transduced cells were rested for 2 days, then puromycin was added for 2 days. Next, cells were seeded for cell viability or clonogenicity assays.

## Additional files

Additional file 1: Supplementary Figures. **Figure S1.** Coverage of the sgRNA library in HCC-827 and CHP-212 cells. a) Coverage of the sgRNA library by deep sequencing at indicated time points for the HCC-827 cell line. Each time point was measured in duplicates and average percentages are represented here. b) Same as in a) but CHP-212 cell line is used instead. **Figure S2.** Principal Components Analysis (PCA) plots for different time points in HCC-827 and CHP-212 cells. PCA plots representing the sgRNA counts obtained for both cell lines. **Figure S3.** Histogram plots of the difference between the maximum and the minimum sgRNA fold changes per gene. These histogram plots show the variability among sgRNAs targeting the same gene calculated as the delta between the maximum and the minimum sgRNA fold changes for each gene for the HCC-827 and the CHP-212 cell lines on day 14. **Figure S4.** Depleted sgRNAs for kinases in HCC-827 and CHP-212 cells. Time points were measured in duplicates and median fold changes are represented here. Dark green colored dots represent the 1 000 non-targeting control sgRNAs and grey colored dots represent the 57 096 targeting sgRNAs. a-d) Scatter plots of fold changes compared to the control time point are shown for the HCC-827 and the CHP-212 cell line. f,g) Scatter plots of fold changes of 57 096 targeting sgRNAs of the HCC-827 and CHP-212 cell lines at indicated time points. e, f) Scatter plot of fold changes for independent replicates at time point day 14 for HCC-827 (S4e) and CHP-212 (S4f), respectively. g,h) Scatter plots of fold changes of 57 096 targeting sgRNAs of the HCC-827 and CHP-212 cell lines at indicated time points. **Figure S5.** Estimation of off-target effects. a) Scatter plots of mRNA expression levels expressed in FPKMs were compared to depletion by Q1 for the HCC-827 cell line. Of the 5 % most depleted genes which is equal to 1 450 sgRNAs, more than 90 % were expressed with a FPKM > 1. b) Of the 1 462 most depleted sgRNAs (5 %) for the CHP-212 cell line, more than 87,5 % were expressed with a FPKM >1. **Figure S6.** MEK inhibitor MEK162 and EGFR inhibitor Erlotinib affected cell viability of the respective cell lines. a,b) HCC-827 and CHP-212 cells were treated with indicated concentrations of Erlotinib (a) or MEK162 (b) and analyzed as described in M&M part. **Figure S7.** Cytogenetics of HCC-827 and CHP-212 cell lines. (PDF 1451 kb) Additional file 2: Table S1. Correlation of sgRNA library distribution between technical replicates in HCC-827 and CHP-212 cells. (XLSX 28 kb) Additional file 3: Table S2. Correlation of sgRNA library distribution between technical replicates of HCC-827 and CHP-212 cells. (XLSX 30 kb) Additional file 4: Table S3. First 1 000 most depleted genes for HCC-827 cell lines. This table shows the list of 1 000 most depleted genes in the HCC-827 cells along with their sgRNA fold changes at the days 14, 21 and 28. Gene expression of target genes is indicated as average FPKMs across 3 replicates. Cumulative scores are calculated as the sum of log2 FC scores obtained at either two or all three time points. Genes are ranked by the cumulative score obtained at all three time points (e.g. as the sum of log FC scores at days 14, 21 and 28 sorted in ascending order). Essential genes identified in recent genetic screens are reported. Genes expressed at low levels in the parental cell line (FPKM < 1) are highlighted in grey. (XLSX 127 kb) Additional file 5: Table S4. First 1 000 most depleted genes for CHP-212 cell lines. This table shows the list of 1 000 most depleted genes in the CHP-212 cells along with their sgRNA fold changes at the days 14, 21 and 28. Gene expression of target genes is indicated as average FPKMs across 3 replicates. Cumulative scores are calculated as the sum of log2 FC scores obtained at either two or all three time points. Genes are ranked by the cumulative score obtained at all three time points (e.g. as the sum of log FC scores at days 14, 21 and 28 sorted in ascending order). Essential genes identified in recent genetic screens are reported. Genes expressed at low levels in the parental cell line (FPKM < 1) are highlighted in grey. (XLSX 130 kb) Additional file 6: Table S5. mRNA expression levels for kinases of top the 20 list for HCC-827 cells. Expression of candidate genes was found to be at very high level in the EGFR-mutant HCC-827 parental line, e.g. 128.09 FPKMs for EGFR. (XLSX 38 kb) Additional file 7: Table S6. mRNA expression levels for kinases of top the 20 list for CHP-212 cells. Several members of the RAS pathway from the top-ranking hits were expressed at high levels in the parental NRAS-mutant CHP-212 line, e.g. 12.03, 3.86 and 15.93 FPKMs for MAP2K1, MAP2K6 and RAF-1 respectively. (XLSX 38 kb) Additional file 8: Table S7. Top hits for the CHP-212 cell line including genes that are not essential, not present in the top 1 000 for the HCC-827 line, and ranked based on their cumulative screening score and expression level. (XLSX 48 kb) Additional file 9: Table S8. Top hits for the CHP-212 cell line including genes that are not essential, not present in the top 1 000 for the HCC-827 line, and ranked based on their cumulative screening score and expression level. (XLSX 45 kb)

## Abbreviations

CRISPR: Clustered regularly interspaced short palindrome repeats
sgRNA: Single guide RNA
DSB: Double-strand breaks
NHEJ: Nonhomologous end-joining
indels: Insertions and deletions
EGFR: Epidermal growth factor receptor
