# Supplementary material for: Identification of oncogenic driver mutations by genome-wide CRISPR-Cas9 dropout screening
Source: BMC Genomics. 2016 Sep 9;17(1):723. doi: 10.1186/s12864-016-3042-2 (PMC5016932; doi:10.1186/s12864-016-3042-2)
Supplement: Additional file 1: — Supplementary Figures. Figure S1. Coverage of the sgRNA library in HCC-827 and CHP-212 cells. a) Coverage of the sgRNA library by deep sequencing at indicated time points for the HCC-827 cell line. Each time point was measured in duplicates and average percentages are represented here. b) Same as in a) but CHP-212 cell line is used instead. Figure S2. Principal Components Analysis (PCA) plots for different time points in HCC-827 and CHP-212 cells. PCA plots representing the sgRNA counts obtained for both cell lines. Figure S3. Histogram plots of the difference between the maximum and the minimum sgRNA fold changes per gene. These histogram plots show the variability among sgRNAs targeting the same gene calculated as the delta between the maximum and the minimum sgRNA fold changes for each gene for the HCC-827 and the CHP-212 cell lines on day 14. Figure S4. Depleted sgRNAs for kinases in HCC-827 and CHP-212 cells. Time points were measured in duplicates and median fold changes are represented here. Dark green colored dots represent the 1 000 non-targeting control sgRNAs and grey colored dots represent the 57 096 targeting sgRNAs. a-d) Scatter plots of fold changes compared to the control time point are shown for the HCC-827 and the CHP-212 cell line. f,g) Scatter plots of fold changes of 57 096 targeting sgRNAs of the HCC-827 and CHP-212 cell lines at indicated time points. e, f) Scatter plot of fold changes for independent replicates at time point day 14 for HCC-827 (S4e) and CHP-212 (S4f), respectively. g,h) Scatter plots of fold changes of 57 096 targeting sgRNAs of the HCC-827 and CHP-212 cell lines at indicated time points. Figure S5. Estimation of off-target effects. a) Scatter plots of mRNA expression levels expressed in FPKMs were compared to depletion by Q1 for the HCC-827 cell line. Of the 5 % most depleted genes which is equal to 1 450 sgRNAs, more than 90 % were expressed with a FPKM > 1. b) Of the 1 462 most depleted sgRNAs (5 %) for the CHP-212 cell li [file 12864_2016_3042_MOESM1_ESM.pdf]

Supplementary figures

a

HCC-827 $EGFR^{delE746}$

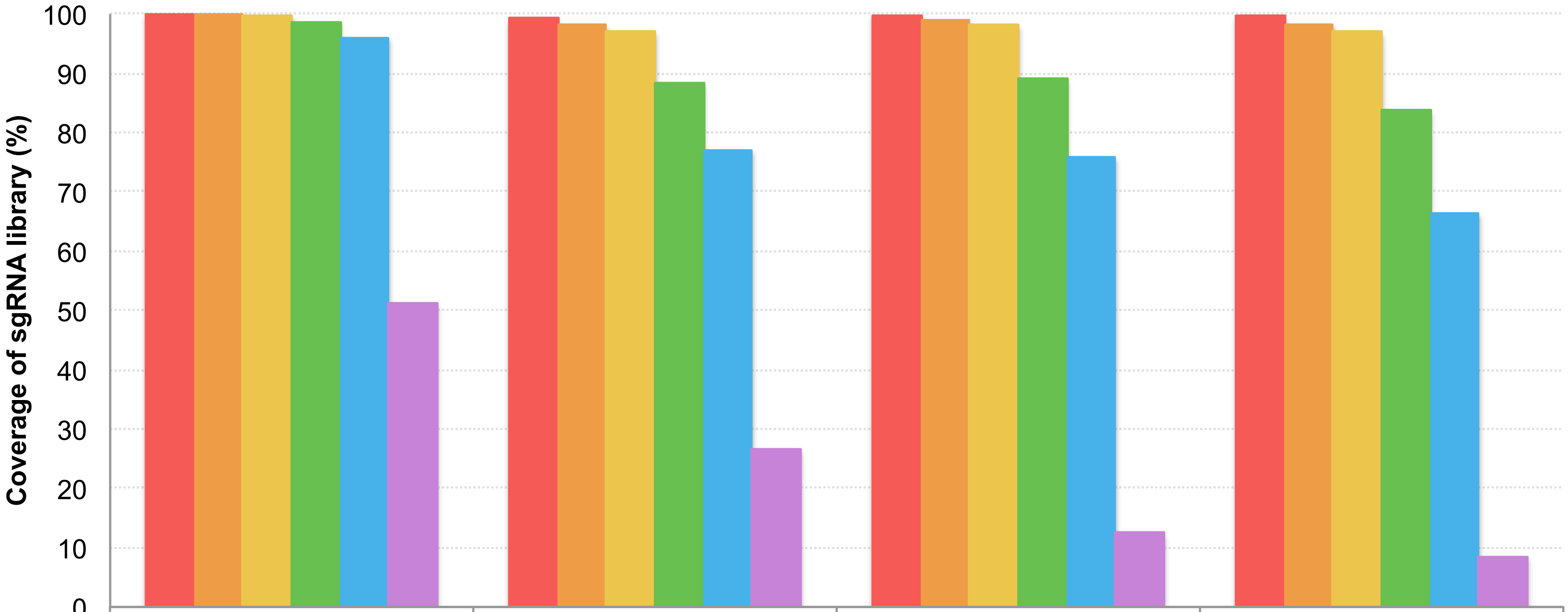

	Control (Day -10)	Day 14	Day 21	Day 28
≥1	100.0	99.2	99.8	99.5
>5	99.9	98.2	99.0	98.3
>10	99.9	97.1	98.1	96.9
>50	98.8	88.3	89.1	83.7
>100	96.0	77.1	75.8	66.4
>500	51.4	26.7	12.5	8.5

Kiessling MK – Supplementary Figure 1

b

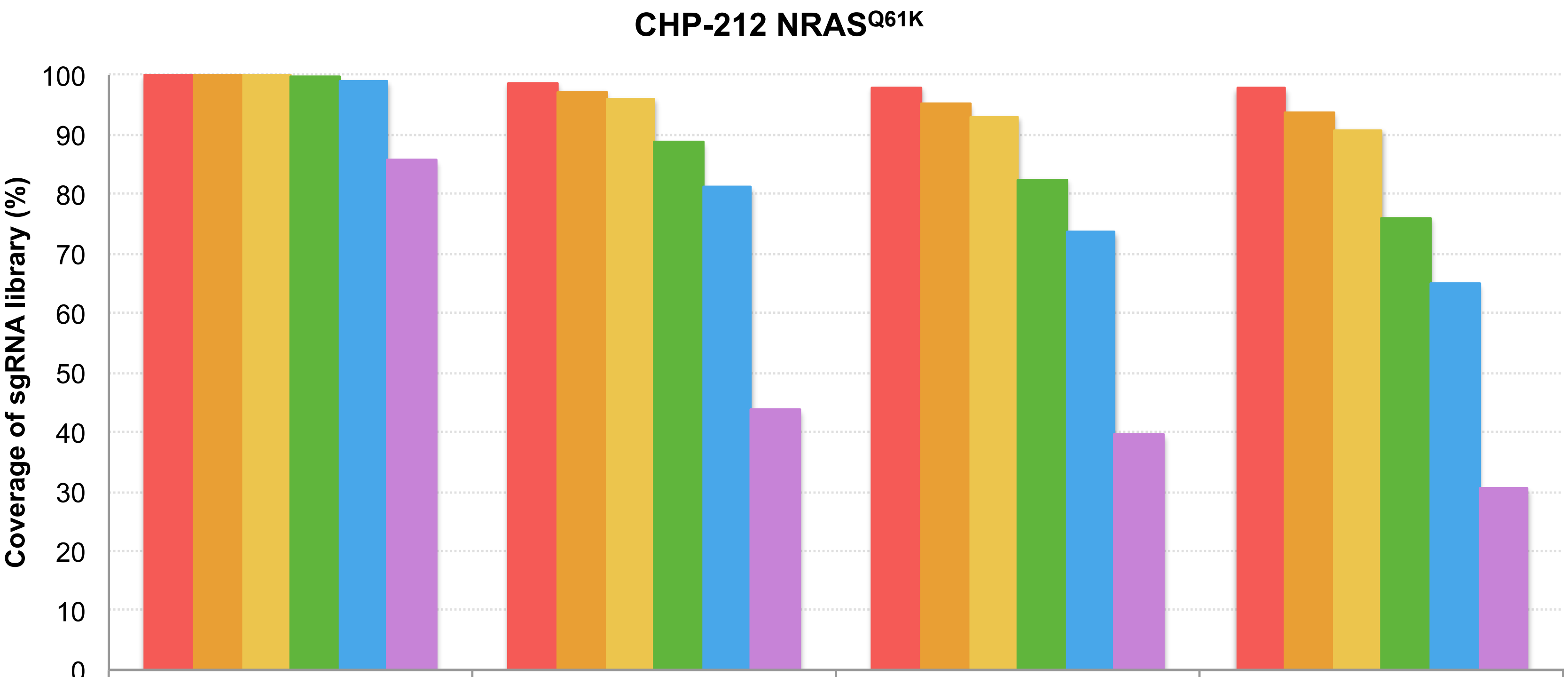

	Control (Day -10)	Day 14	Day 21	Day 28
≥1	100.0	98.7	98.0	97.7
>5	100.0	97.3	95.1	93.6
>10	100.0	96.0	93.0	90.8
>50	99.6	88.6	82.5	76.1
>100	98.9	81.0	73.6	64.8
>500	85.6	43.7	39.5	30.5

Kiessling MK – Supplementary Figure 1

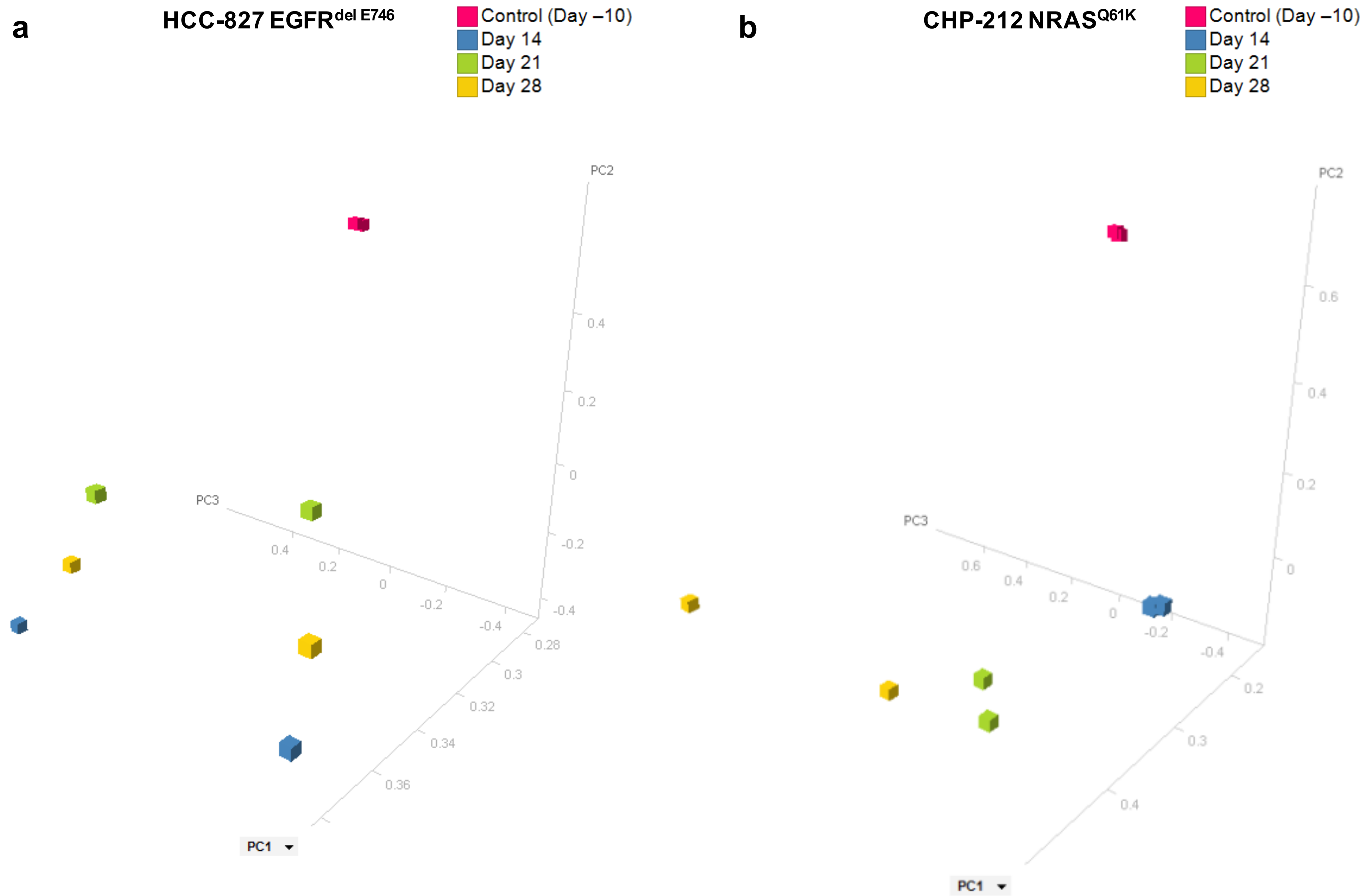

Kiessling MK – Supplementary Figure 2

a

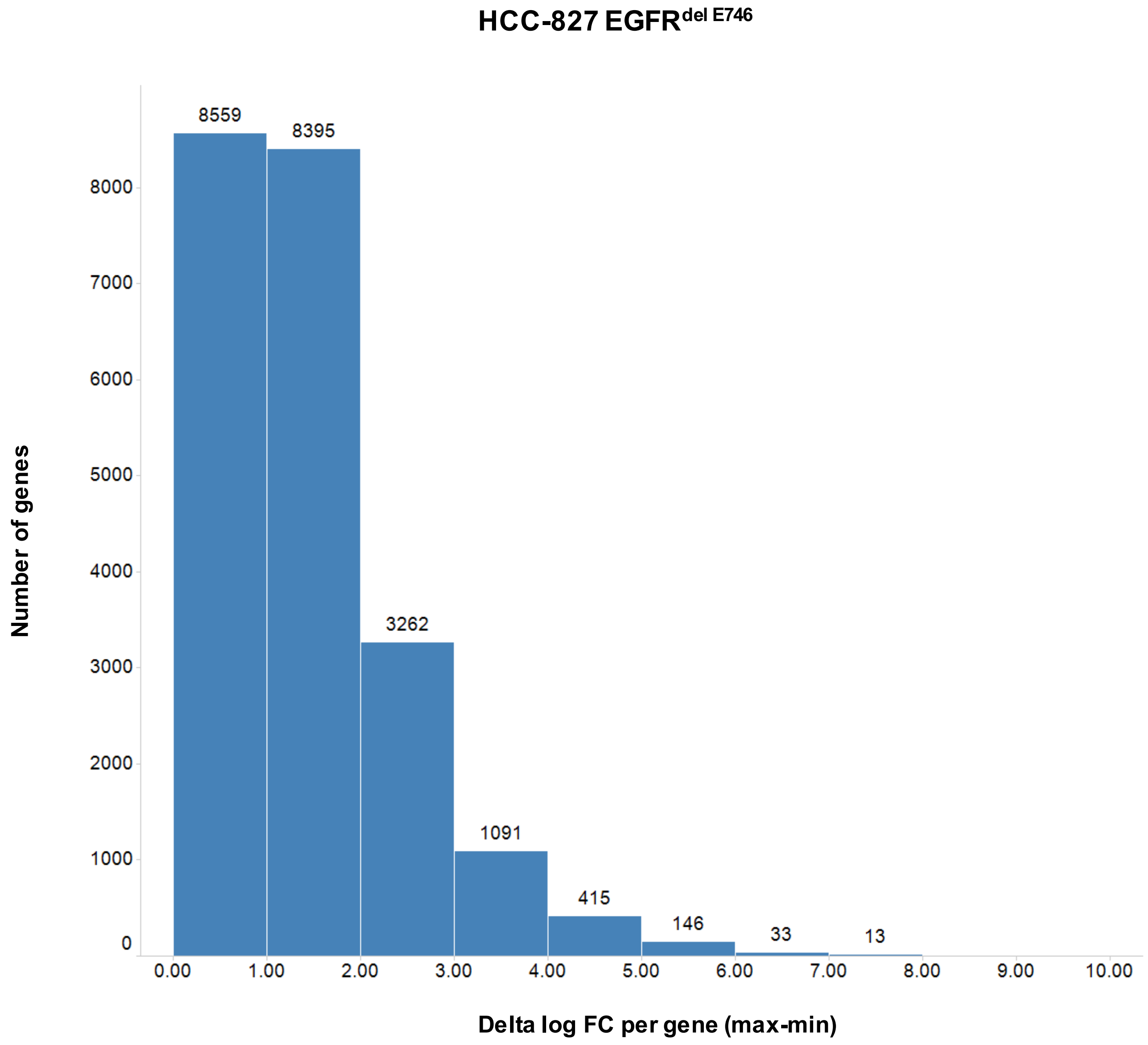

Kiessling MK – Supplementary Figure 3

b

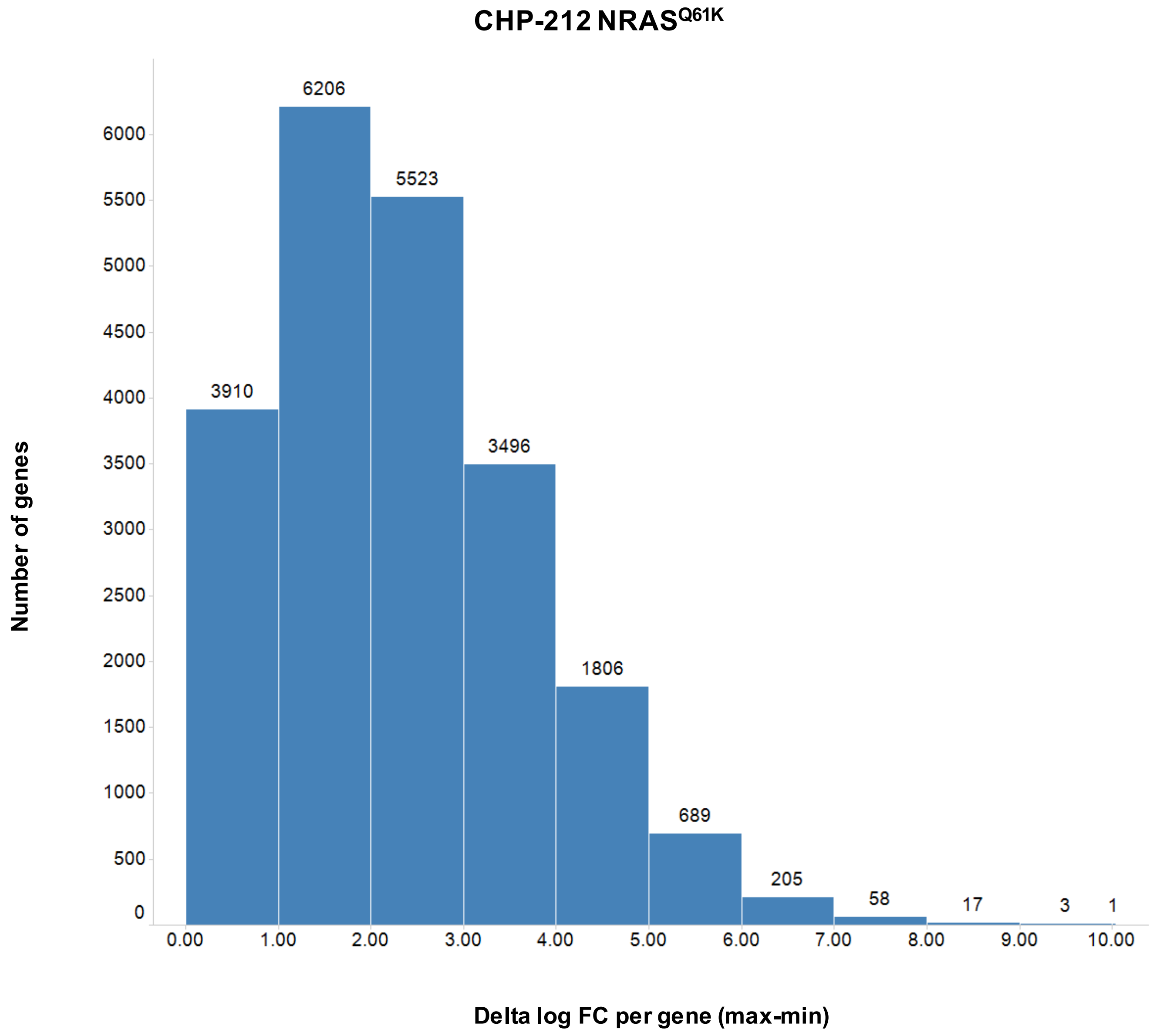

Kiessling MK – Supplementary Figure 3

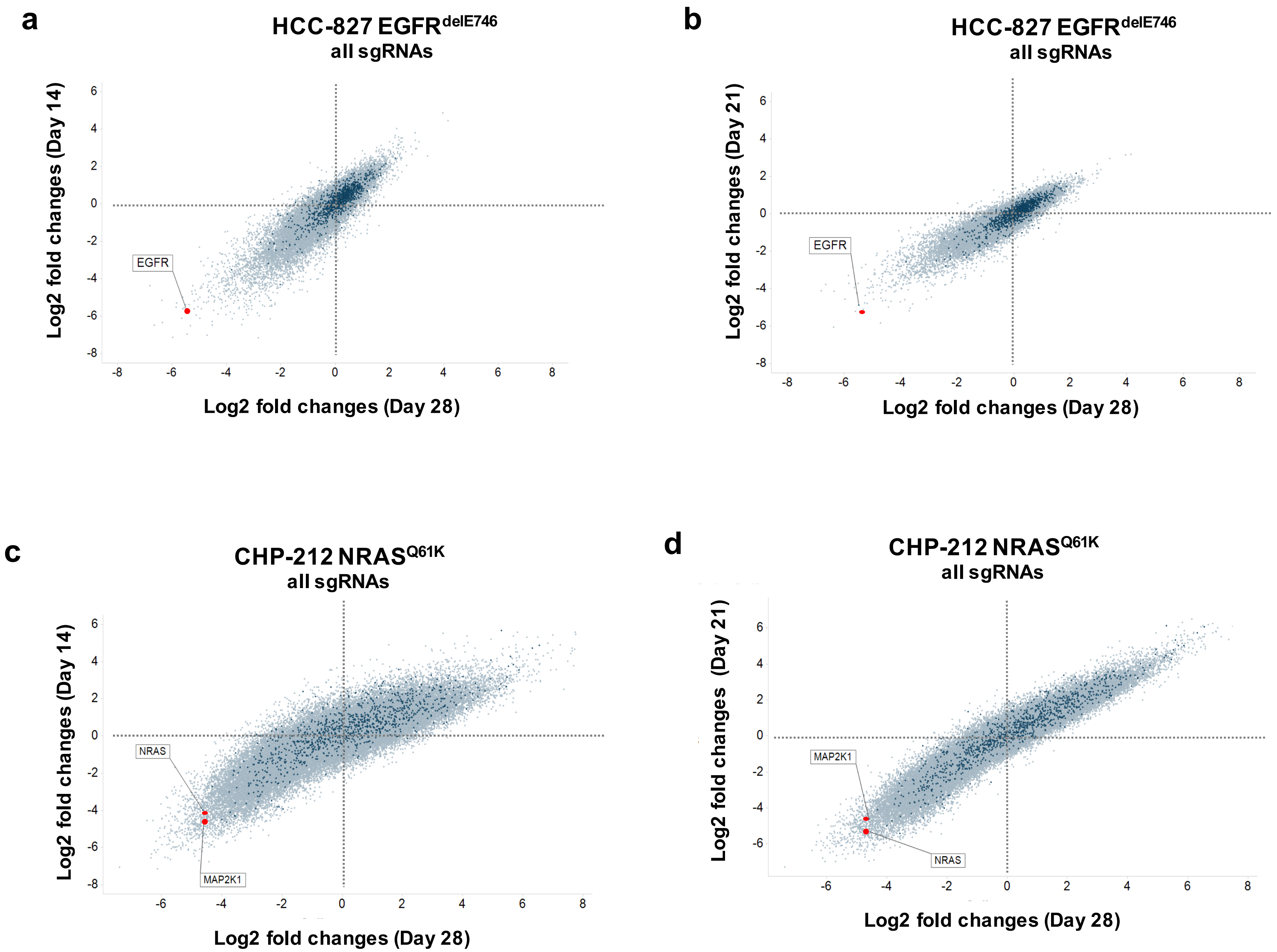

Kiessling MK – Supplementary Figure 4

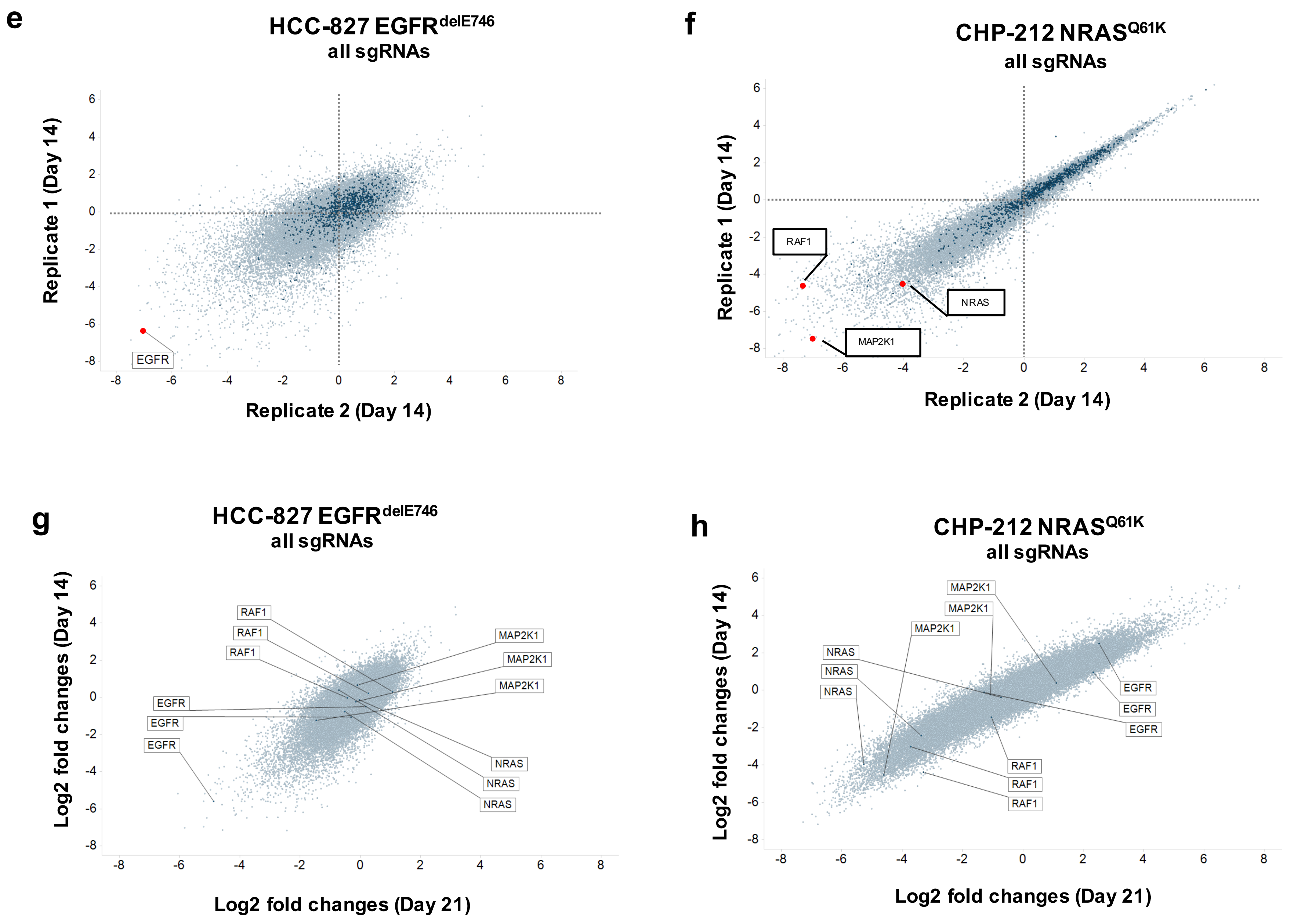

Kiessling MK – Supplementary Figure 4

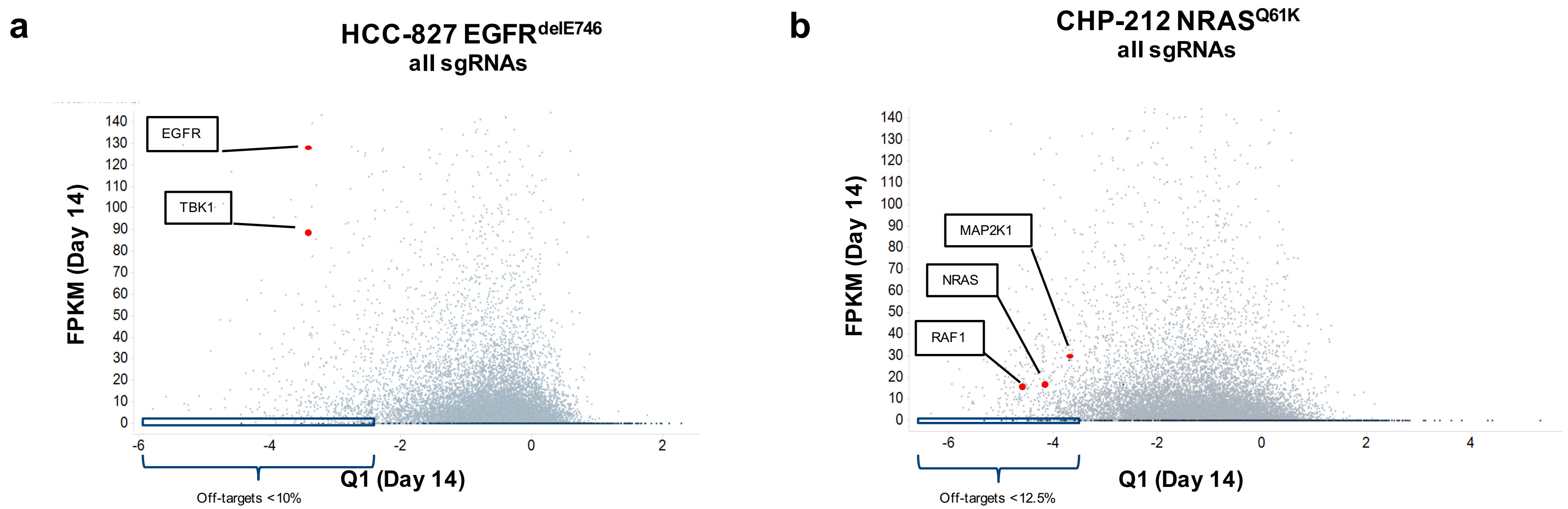

Kiessling MK – Supplementary 5

a

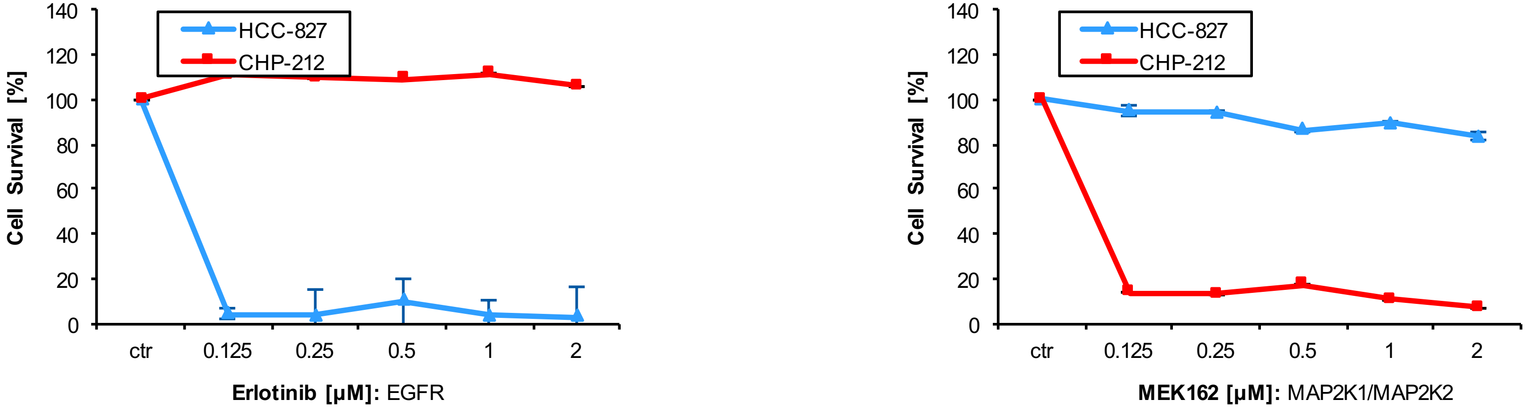

b

Kiessling MK – Supplementary Figure 6

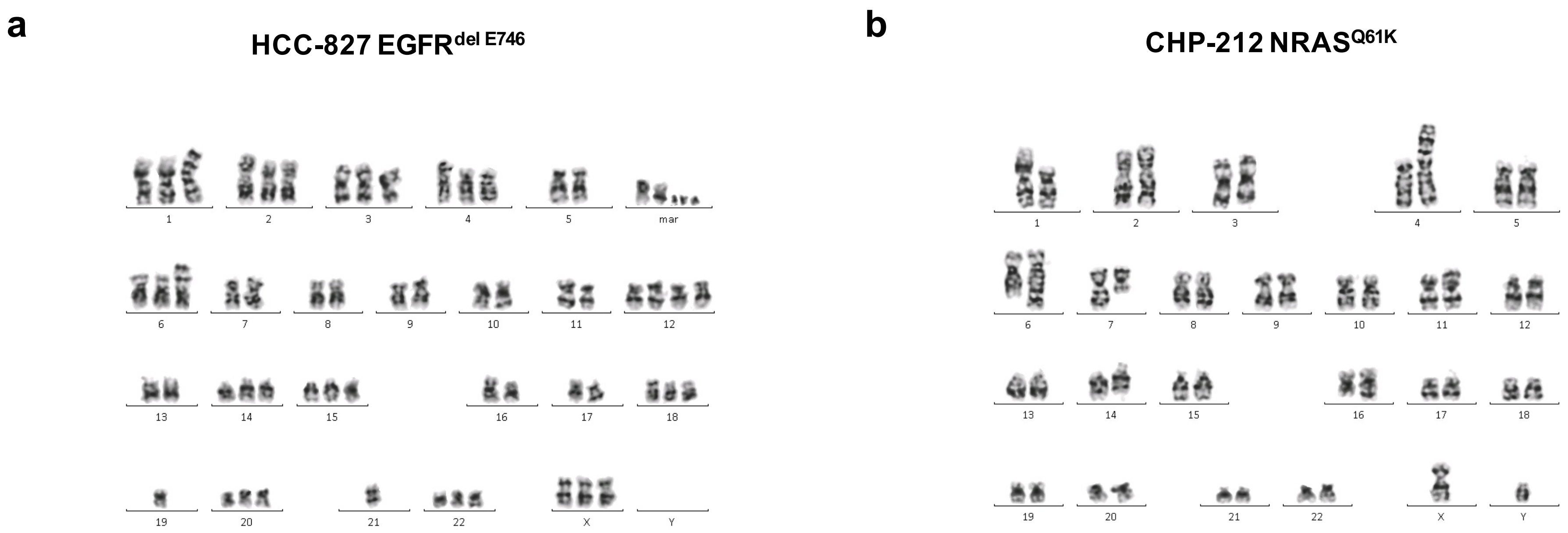

Kiessling MK – Supplementary Figure 7
